# Emotional Dysregulation: Epidemiology and Genetic Features from Childhood towards Adulthood

**DOI:** 10.1192/j.eurpsy.2022.135

**Published:** 2022-09-01

**Authors:** M. Nobile

**Affiliations:** Scientific Institute IRCCS ’E. Medea’, Child Psychiatry Department, Bosisio Parini, Italy

**Keywords:** gene-environment interaction, emotional dysregulation, Developmental trajectories, Methylation

## Abstract

Emotional dysregulation (ED) is a dimensional, transdiagnostic domain that is associated with multiple categorical psychiatric diagnoses from childhood to adulthood, representing a risk for increased problems in affect, behavior, and cognition [1]. Traditionally, the nature of ED trait has been studied with top down approaches: quantitative evaluation of ED is possible through “Dysregulation Profile” scoring, which is measured through composite scales of the “Achenbach System of Empirically Based Assessment” (ASEBA) [2] questionnaires. Dysregulation profile is characterized by severe anxiety and affective symptoms, impulsive and/or aggressive behaviours and metacognitive difficulties. More recently, different researchers also applied bottom up approaches to evaluate the presence of ED in both general population and clinically referred samples [3]. Also in these cases, the results showed that ED is a trait, stable through time and across different cultures and societies, associated with higher presence of psychiatric diagnosis. It is important to note that these non-traditional statistical approaches highlighted that, in adulthood, ED is characterized by elevated scores in both externalizing and internalizing areas. In this contribution, the research aimed at disentangling the etiology of ED, which is crucial to treat and prevent worst evolution associated with this trait, will be revised. Many efforts have been done to understand the complex interaction between genetic and environmental risk factors which predispose patients to develop and maintain ED. [1] Aitken, et al. (2019). JAD, 253, 87-95. [2] Achenbach & Rescorla (2001). Manual for the ASEBA school-age forms and profiles. [3] Bianchi, et al (2017). ECAP, 26(5), 549-557.

**Disclosure:**

No significant relationships.

